# A Drug Screening Method Based on the Autophagy Pathway and Studies of the Mechanism of Evodiamine against Influenza A Virus

**DOI:** 10.1371/journal.pone.0042706

**Published:** 2012-08-10

**Authors:** Jian-Ping Dai, Wei-Zhong Li, Xiang-Feng Zhao, Ge-Fei Wang, Jia-Cai Yang, Lin Zhang, Xiao-Xuan Chen, Yan-Xuan Xu, Kang-Sheng Li

**Affiliations:** 1 Department of Microbiology and Immunology, Shantou University Medical College, Shantou, Guangdong, China; 2 Department of Veterinary Medicine, University of Maryland, College Park, Maryland, United States of America; Salute San Raffaele University School of Medicine, Italy

## Abstract

In this research, we have established a drug screening method based on the autophagy signal pathway using the bimolecular fluorescence complementation - fluorescence resonance energy transfer (BiFC-FRET) technique to develop novel anti-influenza A virus (IAV) drugs. We selected *Evodia rutaecarpa* Benth out of 83 examples of traditional Chinese medicine and explored the mechanisms of evodiamine, the major active component of *Evodia rutaecarpa* Benth, on anti-IAV activity. Our results showed that evodiamine could significantly inhibit IAV replication, as determined by a plaque inhibition assay, an IAV vRNA promoter luciferase reporter assay and the Sulforhodamine B method using cytopathic effect (CPE) reduction. Additionally, evodiamine could significantly inhibit the accumulation of LC3-II and p62, and the dot-like aggregation of EGFP-LC3. This compound also inhibited the formation of the Atg5-Atg12/Atg16 heterotrimer, the expressions of Atg5, Atg7 and Atg12, and the cytokine release of TNF-α, IL-1β, IL-6 and IL-8 after IAV infection. Evodiamine inhibited IAV-induced autophagy was also dependent on its action on the AMPK/TSC2/mTOR signal pathway. In conclusion, we have established a new drug screening method, and selected evodiamine as a promising anti-IAV compound.

## Introduction

Influenza A virus (IAV) poses a global health and economic threat as the next worldwide pandemic. Current antiviral drugs are limited by their negative side effects and by the emergence of drug-resistant strains [1]. Current vaccine production is also still problematic due to the difficulty of working with the relatively low immunogenicity of some strains and the need to protect against the large number of strains circulating in the environment [2], [3], [4], [5]. Therefore, it is still urgent to develop novel drug screening methods. It has been reported that macroautophagy (hereafter referred to as autophagy) is involved in the replication of IAV, and inhibiting autophagy will inhibit the replication of IAV [6], and recently, Ma J. et al have shown that the highly pathogenic avian influenza viruses (H5N1) can cause “autophagic cell death” through suppressing mTOR (mammalian target of rapamycin) signaling, and inhibition of autophagy can reduce H5N1-mediated cell death and mortality [7]. So autophagy inhibition is a good strategy for developing novel anti-IAV drugs.

Autophagy is a highly conserved process in all eukaryotic cells, the regulation of autophagy is complicated, it can be divided into six stages: initiation, nucleation, elongation, closure, maturation and degradation [8]. Initiation of autophagy is regulated by mTORC1 via ULK1/2-Atg13- FIP200-Atg101 complex [9], [10]. At initiation, the mTORC1 complex is further negatively regulated by the tuberous sclerosis complex (TSC) 1/2 heterodimer via promoting the conversion of Rheb-GTP to Rheb-GDP. TSC2 is further regulated by PI3K/AKT, LKB1/AMPK and MAPK pathways. The activated AMPK can phosphorylate and activate TSC2, and sequentially inhibit mTOR and activate autophagy [11].

During nucleation and maturation stages, Beclin1 plays a central role via its interactions with many cofactors (Atg14L, UVRAG, Bcl2, Bif-1, Rubicon, Ambra1, HMGB1, nPIST, VMP1, SLAM, IP3R, PINK and Survivin). Beclin1 complex I (Vps34, p150, Beclin1 and Atg14) is essential for autophagosome formation, Beclin1 complex II (Vps34, p150, Beclin1 and UVRAG) promotes the fusion of autophagosomes with lysosomes [12], [13]. Beclin1 has been proved to be a major target for manipulation of autophagy by many viruses [14]. But because many proteins interact with Beclin1, the regulation of Beclin1 is very complicated.

During elongation stage, there is an important event, the formation of the Atg5-Atg12/Atg16 heterotrimer [15]. Comparing with other complexes, such as ULK1/2-Atg13- FIP200-Atg101 complex, Beclin1 complex I and II, the regulation of the Atg5-Atg12/Atg16 heterotrimer is relatively more simple and explicit. As shown in **Fig. S1**, at elongation stage, Atg12 must conjugate with Atg5, mediated by Atg7 and Atg10. Subsequently, this Atg5-Atg12 heterodimer must conjugate with Atg16 to form an Atg5-Atg12/Atg16 heterotrimer, which is necessary to promote the transformation of LC3-I (Atg8) to LC3-II (Atg8-PE), LC3-II is necessary for the formation of autophagosome. This process can be reversed by Atg4. Moreover, Nao Jounai et al. have shown that the Atg5–Atg12 conjugate plays an important role in innate antiviral immune responses against viral infection, and inhibiting the formation of Atg5–Atg12 heterodimer can inhibit virus replication [16]. So we select the formation of Atg5-Atg12/Atg16 heterotrimer as our drug screening target and establish a drug screening method using the bimolecular fluorescence complementation- fluorescence resonance energy transfer (BiFC-FRET) technique.

BiFC-FRET is a recently emerged technique and is used widely in biology, medicine and chemistry. The BiFC technique can investigate the interaction of two proteins in live cells, and the BiFC-FRET technique can investigate the interaction of three proteins in live cells [17]. To the authors’ knowledge, no report describing the use of the BiFC-FRET technique to construct a drug screening method has been published.

Among traditional Chinese medicines, there are many antiviral drugs whose mechanism of action is only poorly understood. Using our drug screening method, we screened 83 types of traditional Chinese medicines, and found *Evodia rutaecarpa* Benth had the highest activity, so we selected *Evodia rutaecarpa* Benth as our medicinal plant of interest. We purchased evodiamine, the major active component of *Evodia rutaecarpa* Benth, and determined its antiviral activity to check the availability of our drug screening method, and further explored the mechanism of evodiamine action on anti-IAV activity.

## Results

### The Design of our Drug Screening Method and the Results of the Initial Screening

The design of our drug screening method was shown in **Fig. S2**. Human Atg5 and Atg12 genes were fused with the N′- and C′- fragments of a red fluorescence protein (RFP), respectively. The Atg16 was inserted into a pEGFP-C1 plasmid. After cotransfection with these three plasmids, pMC-atg5, pMN-atg12 and pEGFP-atg16, an intact RFP would reconstitute due to the interaction between Atg5 and Atg12, this process was called BiFC, sequentially, the FRET would take place due to the interaction between the Atg5-Atg12 heterodimer and Atg16. We determined the influence of the drugs on the interaction between Atg5, Atg12 and Atg16 using a microplate reader at 610 and 509 nm after excitation at 488 nm. The FRET efficiency (FRET^e^) was expressed as the ratio of acceptor (610 nm)- and the donor (509 nm)-emission intensities after the deduction of the background intensity. During initial screening, we did not quantify the interaction between Atg5 and Atg12, because the influence of drugs on the formation of Atg5-Atg12 heterodimer finally influenced the formation of the Atg5-Atg12/Atg16 heterotrimer. But from the color graphs obtained from an upright fluorescence microscope (Nikon Eclipse 90i), the changes in each treatment group could be observed.

Using this screening method, we screened 83 types of traditional Chinese medicines, and the result was shown in **Table S1**, there were 15 examples that could significantly (*P*<0.05) inhibit the formation of the Atg5-Atg12/Atg16 heterotrimer, i.e. *Serissa serissoides* (DC.) Druce., *Peucedanum praeruptorum* Dunn., *Aloe barbadensis* Miller., *Borneolum syntheticum, Ginkgo biloba* L., *Schizonepeta tenuifolia* Briq., *Hedyotis diffusa* Willd., *Rheum palmatum* L., *Litsea cubeba (Lour.)* Pers., *Kummerowia striata* (Thunb.) Schindl., *Apium graveolens* L. var. dulce DC., *Chrysanthemum indicum* L., *Scutellaria baicalensis* Georgi., *Coptis chinensis* Franch., *Silybum marianum* L. In addition, there were 5 examples that could significantly (*P*<0.01) inhibit the formation of the Atg5-Atg12/Atg16 heterotrimer, i.e. *Areca catechu* L., *Buddleja lindleyana* Fort., *Eugenia caryophyllata* Thunb., *Curcuma longa* L., *Evodia rutaecarpa* Benth. In these medicinal plants, to our knowledge, there were 7 examples that had not been reported to possess anti-IAV activity, i.e. *Peucedanum praeruptorum* Dunn., *Borneolum syntheticum, Ginkgo biloba* L., *Silybum marianum* (L.) Gaertn., *Buddleja lindleyana* Fort., *Eugenia caryophyllata* Thunb., and *Evodia rutaecarpa* Benth, and in these 7 medicinal plants, the crude extract of *Evodia rutaecarpa* Benth showed the highest activity, so we chose *Evodia rutaecarpa* Benth as our medicinal plant of interest, and purchased evodiamine (Evo), the major active component of *Evodia rutaecarpa* Benth, detected the anti-IAV activity of evodiamine and checked the availability of our drug screening method, meanwhile, we also explored the mechanisms of evodiamine on anti-IAV activities.

Moreover, we also determined the Z′-factor which was a statistical parameter to quantify the suitability of a particular assay for use in a high-throughput screen, the Z′-factor of our drug screening method was 0.5153. According to the criterion stated in the previous report [18], our drug screening method had a valid Z′ value (Z′>0.5).

### Evodiamine also Inhibits the Formation of the atg5-atg12/atg16 Heterotrimer Determined by BiFC-FRET Technique and Co-immunoprecipitation Assay

As above mentioned, evodiamine is the major active component of *Evodia rutaecarpa* Benth, its molecular formula is C_19_H_17_N_3_O, its molecular mass is 303.23. In the initial screening, Crude extract of *Evodia rutaecarpa* Benth could significantly (*P*<0.01) inhibit the formation of the Atg5-Atg12/Atg16 heterotrimer. Here we identified whether evodiamine also had this activity. Purity of evodiamine we purchased was>98%.

**Figure 1:**
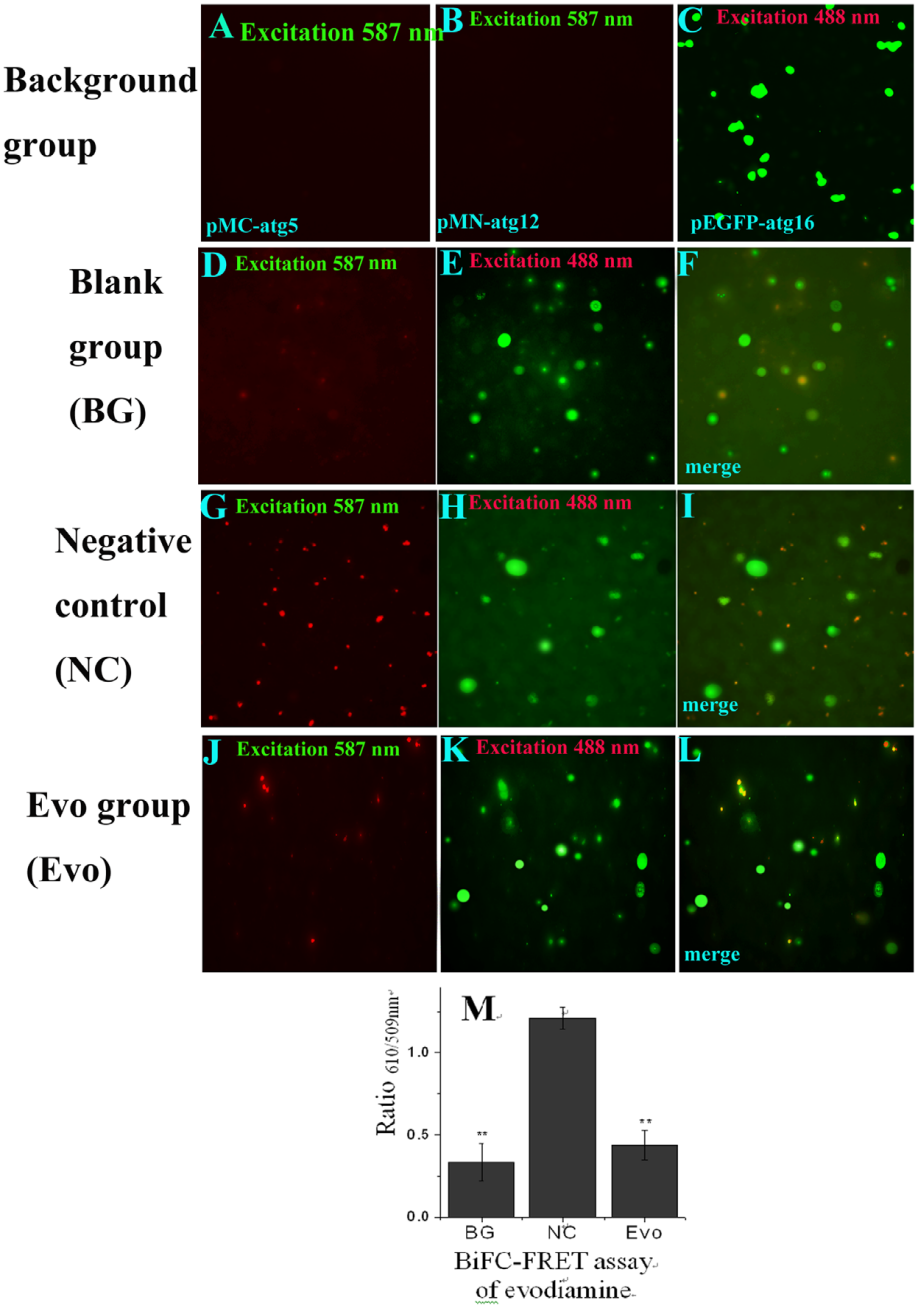
Evodiamine inhibits the interaction of Atg5, Atg12 and Atg16 determined by the BiFC-FRET assay. (**A**, **B**) The A549 cells were transfected with pMC-atg5 or pMN-atg12, respectively. (**C**) The A549 cells were transfected with the pEGFP-atg16 plasmid and treated with evodiamine (12.5 µg/mL). (**D–L**) The BiFC-FRET assay of evodiamine, the A549 cells in all groups were cotransfected with the pMC-atg5, pMN-atg12 and pEGFP-atg16 plasmids. In the BG group, the cells were not infected and not treated with drugs. In NC and Evo groups, the cells were infected (MOI = 2.0), and at the same time treated without or with evodiamine (12.5 µg/mL) for 8 h, respectively. The graphs were obtained using an upright fluorescence microscope (10×40). The **D,** **G** and **J** graphs were obtained after excitation at 587 nm to show the BiFC signal between atg5 and atg12. The **E,** **H** and **K** graphs were obtained after excitation at 488 nm, but the FRET signal was determined at 610 nm and 509 nm after excitation at 488 nm using a microplate reader, and the result of FRET assay was shown in (**M**). The **F**, **I** and **L** showed the colocalization of atg5, atg12 and atg16. Data shown were the mean±SD of two independent experiments with three replicates. *P<0.05, **P<0.001, *vs.* the NC.

As shown in **Fig. 1** **,** in the background groups (**Fig. 1** **A and B**), A549 cells were transfected with the pMC-atg5 or pMN-atg12 plasmid, and showed no BiFC signal because the individual N′- or C′- fragments of red fluorescence protein could not emit red light after excitation. In **Fig. 1** **C**, A549 cells were transfected with a pEGFP-atg16 plasmid and treated these cells with evodiamine (12.5 µg/mL). The result showed that the CMV promoter of the plasmid we used could normally transcript under the treatment of evodiamine (12.5 µg/mL). In the blank group (BG), the cells were cotransfected with the pMC-atg5, pMN-atg12 and pEGFP-atg16 plasmids, and did show some BiFC (**Fig. 1** **D**) and FRET (**Fig. 1** **M**) signals, which might be due to the autophagy of the cell *per se*. In the negative control (NC) group, the cells were cotransfected with these three plasmids and infected with IAV A/ShanTou/169/06 (MOI = 2.0, incubation time = 8 h), the BiFC (**Fig. 1** **G**) and FRET (**Fig. 1** **M**) signals dramatically increased, which showed that the formation of the Atg5-Atg12/Atg16 heterotrimer was increased after IAV infection. Evodiamine (12.5 µg/mL) could significantly (*P*<0.01) down-regulate the BiFC (**Fig. 1** **J**) and FRET (**Fig. 1** **M**) signals, which proved evodiamine (12.5 µg/mL) could significantly inhibit the formation of the Atg5-Atg12/Atg16 heterotrimer.

**Figure 2:**
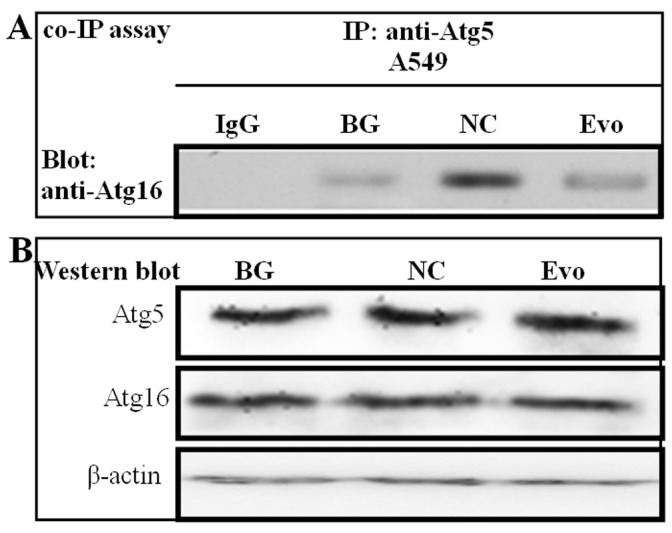
Evodiamine inhibits the interaction of Atg5-Atg12 and Atg16 determined by the Co-immunoprecipitation assay. (**A**) A549 cells in all groups were cotransfected with the pMC-atg5, pMN-atg12 and pEGFP-atg16 plasmids. A549 cell lysate was subjected to immunoprecipitation with an anti-Atg5 antibody, and the precipitates were subjected to Western blotting analysis using anti-Atg16 antibody. Normal rabbit IgG was used as a control. In the blank group (BG), the cells were not infected and not treated with drugs; in the negative control (NC) and Evo-treatment groups (Evo), the cells were infected (MOI = 0.001) and treated without and with evodiamine (12.5 µg/mL) for 24 h, respectively. (**B**) The levels of Atg5, Atg16 and β-actin of the whole cell lysate were determined by Western blotting.

To further confirm the influence of evodiamine on the interaction between the Atg5-Atg12 heterodimer and Atg16 in physiological condition, we performed an immunoprecipitation assay using anti-Atg5 antibody; then performed a Western blot assay using anti-Atg16 antibody. As shown in **Fig. 2** **A**, the conjugation of Atg5 and Atg16 was obviously increased after infection with IAV A/ShanTou/169/06 (NC group comparing with the BG group), and evodiamine could obviously inhibit this process (Evo group comparing with the NC group), which also indicated that evodiamine could inhibit the formation of the Atg5-Atg12/atg16 heterotrimer.

### Evodiamine Inhibits the Replication of IAV

**Figure 3:**
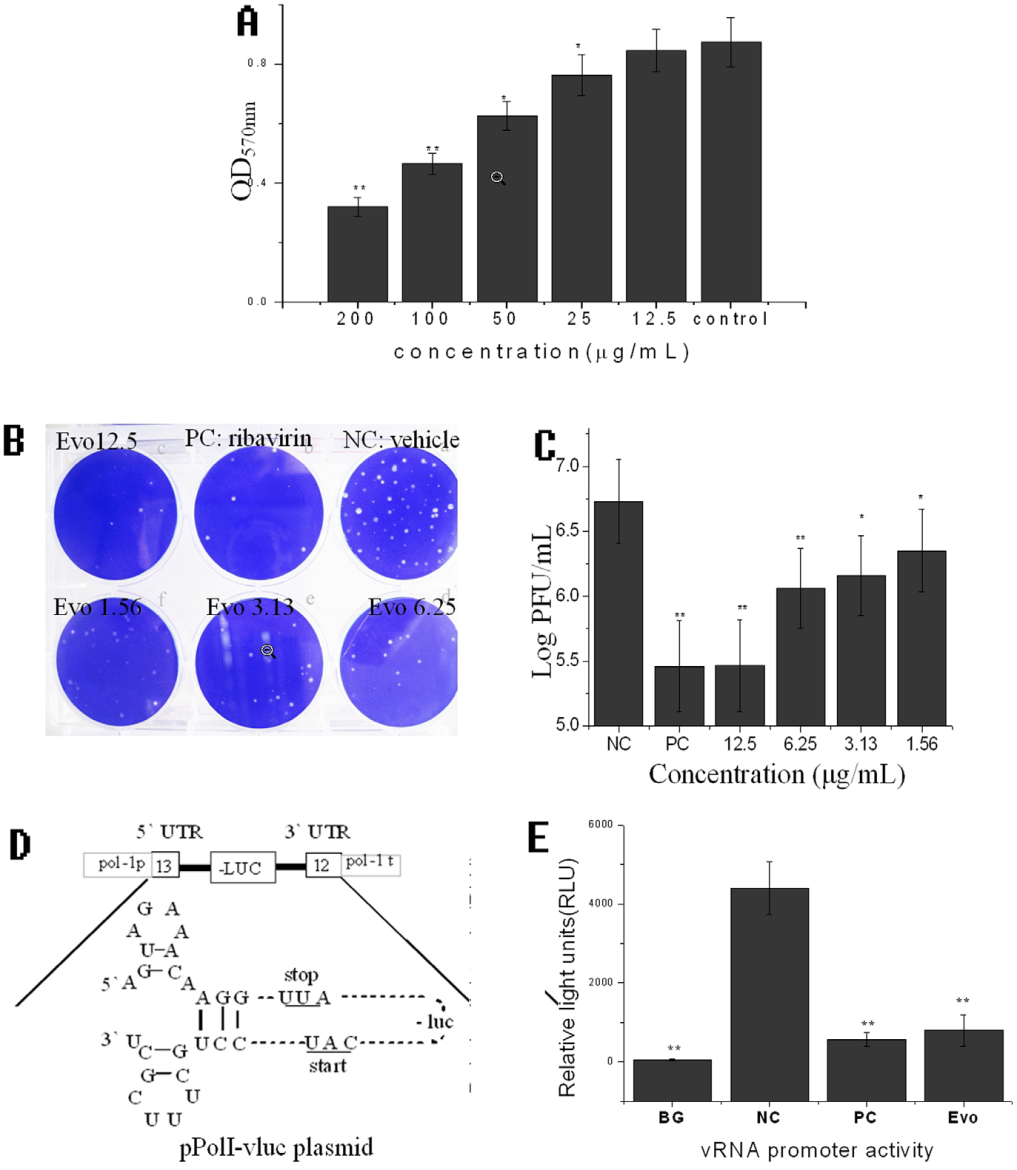
**Evodiamine inhibits the IAV replication.** (**A**) The result of the cytotoxicity assay. The cells in the control group were not treated with evodiamine but solvent vehicle (DMSO <0.5%). (**B**) The plaque inhibition assay of evodiamine. (**C**) The result of the plaque inhibition assay. In the NC group, the cells were infected with IAV, but not treated with drugs; In the PC group, the cells were infected with IAV and treated with ribavirin (25 µg/ml). MOI = 0.001, incubation time = 48 h. (**D**) The schematic representation of the vRNA promoter luciferase reporter (pPolI-vluc) plasmid. (**E**) The inhibition of evodiamine on IAV replication using IAV vRNA promoter luciferase reporter plasmid. In the blank group (BG), the cells were transfected with the pPolI-vluc plasmid but not infected with the IAV; In the NC group, the cells were transfected with the pPolI-vluc plasmid and infected with the IAV, but not treated with drugs; In the PC and Evo groups, the cells were transfected with pPolI-vluc, infected with IAV and treated with ribavirin (25 µg/ml) and evodiamine (12.5 µg/ml), respectively; Data shown were the mean±SD of two independent experiments of three replicates. *P<0.05, **P<0.01, *vs.* the NC.

Before drug screening assay, we had determined the cytotoxicity of our collected plant extracts and compounds on the MDCK and A549 cells. Here we showed the cytotoxicity of evodiamine on MDCK in **Fig. 3** **A**, evodiamine significantly inhibited the viability of MDCK cells in the range of 25 to 200 µg/mL; Y = −0.0028X + 0.8305, R^2^ = 0.9248, IC_50_ = 140.6607 µg/mL. We selected 12.5 µg/mL as the optimal concentration for our experiments.

We first investigated the effect of evodiamine on the replication of IAV A/ShanTou/169/06 using a plaque inhibition assay. As indicated in **Fig. 3** **B** and **C**, evodiamine significantly inhibited viral replication in the range of 1.54–12.5 µg/mL. Y = −1832120 Ln(X) + 3458753.99, R^2^ = 0.98, EC_50_ = 2.6154 µg/mL. The antiviral index (IC_50_/EC_50_) was 53.78.

Moreover, we also investigated the antiviral activity of evodiamine using an IAV vRNA promoter luciferase reporter plasmid. IAV consists of eight gene segments, and each segment is flanked by the untranslated regions (UTRs) at the 5′ and 3′ termini, which are highly conserved and can anneal to form a bulged duplex structure that is essential for transcription and replication, called the vRNA promoter. In our study, we inserted a negative strand luciferase gene into these two UTRs to construct a “-5′UTR- (-luc)- UTR3′-” fragment. This fragment was then flanked by human rRNA promoter and rRNA terminator again to construct a “- hpoI p-5′UTR- (-luc)- UTR3′- hpoI t-” fragment. Finally, this fragment was inserted into a pGL3-Basic plasmid and named pPolI-vluc plasmid (**Fig. 3** **D**). After transfection with this plasmid for 6 h, IAV was added (MOI = 0.01), and the luciferase (Luc) activity was determined at 18 h p.i. The results were shown in **Fig. 3** **E**. Evodiamine (12.5 µg/mL) was found to significantly (*P*<0.05) inhibit the replication of IAV, as comparing with the NC group.

**Figure 4:**
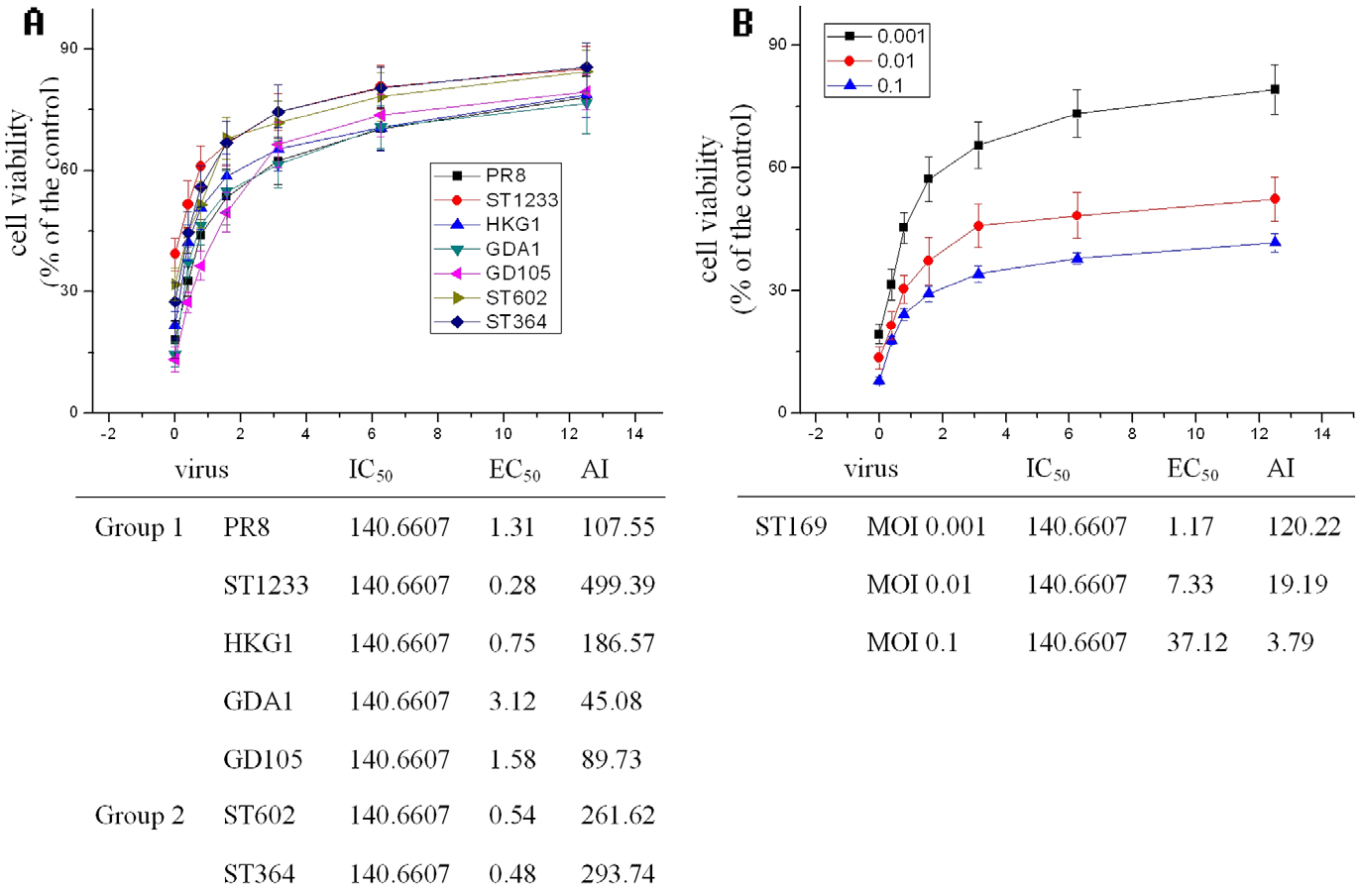
Evodiamine inhibits the replications of several IAV strains. (**A**) The antiviral activity of evodiamine on 7 IAV strains including A/PuertoRico/8/34 (H1N1, PR8), A/ShanTou/1233/06 (H1N1, ST1233), A/Quail/HongKong/G1/97 (H9N2, HKG1), A/Chicken/Guangdong/A1/03 (H9N2, GDA1), A/Chicken/Guangdong/1/05 (H5N1, GD105), A/ShanTou/602/06 (H3N2, ST602) and A/ShanTou/364/05 (H3N2, ST364) by Sulforhodamine B (SRB) method using CPE reduction, MOI = 0.001, incubation time = 48 h. (**B**) The antiviral activity of evodiamine on A/ShanTou/169/06(H1N1) at different MOI (0.001, 0.01, and 0.1) by Sulforhodamine B (SRB) method using CPE reduction, incubation time = 48 h.

In addition, we had determined the antiviral activity of evodiamine on other 7 IAV strains including A/PuertoRico/8/34 (H1N1), A/ShanTou/1233/06 (H1N1), A/Quail/HongKong/G1/97 (H9N2), A/Chicken/Guangdong/A1/03 (H9N2), A/Chicken/Guangdong/1/05 (H5N1), A/ShanTou/602/06 (H3N2) and A/ShanTou/364/05 (H3N2) by Sulforhodamine B (SRB) method using CPE reduction. The result showed that evodiamine could obviously inhibit these IAV strains. At 12.5 µg/ml, the cell viabilities in all groups remained 76.65–85.57% to the mock control, the EC_50_ of evodiamine on these IAV strains were 1.31, 0.28, 0.75, 3.12, 1.58, 0.54 and 0.48 µg/ml, respectively (**Fig. 4** **A**). We also determined the antiviral activity of evodiamine on A/ShanTou/169/06(H1N1) at different MOI (0.001, 0.01 and 0.1), the result showed that evodiamine could obviously inhibit this IAV strain at MOI of 0.001, 0.01 and 0.1, the EC_50_ were 1.17, 7.33 and 37.12 µg/ml, respectively (**Fig. 4** **B**).

### Evodiamine Inhibits the Accumulation of Autophagosome Induced by IAV

**Figure 5:**
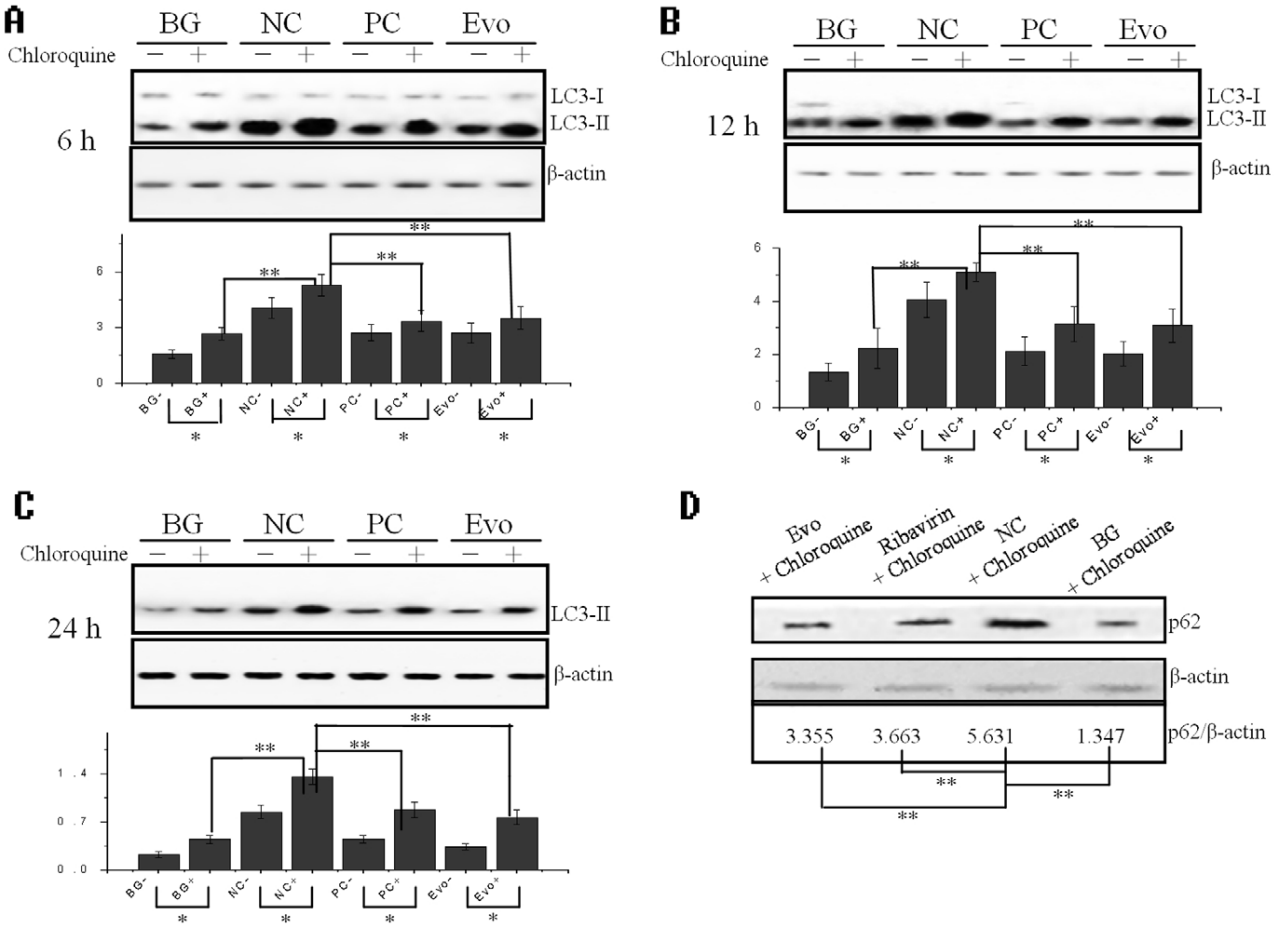
Evodiamine inhibits the IAV-induced autophagy determined by Western blot. (**A–C**) Inhibition of evodiamine on the ratio of LC3-II to β-actin after infection for 6, 12, and 24 h, respectively, as seen by Western blot. MOI = 0.001. (**D**) Inhibition of evodiamine on the ratio of p62 to β-actin after infection for 24 h. In the BG group, the A549 cells were not infected with IAV and not treated with the drugs. In the NC group, the cells were infected with IAV but not treated with the drugs. In the PC and evodiamine (Evo) groups, A549 cells were infected with IAV and treated with ribavirin (25 µg/mL) and evodiamine (12.5 µg/mL), respectively. MOI = 0.001. The total gray value of each band was determined using a Gel-Pro analyzer 6.0. The ratio of LC3-II or p62 to β-actin is presented below. Data shown were the mean±SD of three independent experiments. *P<0.05, **P<0.01.

It has been reported that autophagy significantly increases after IAV infection 2 h, and the ratio of LC3-II to β-actin reaches a large value after IAV infection 6 h [6]. In our study, we first investigated the ratio of LC3-II to β-actin after IAV infection 6, 12 and 24 h, using a Western blot. As shown in **Fig. 5** **A, B** and **C**, after virus infection, the ratio of LC3-II to β-actin in the NC group significantly (*P*<0.01) increased as compared with that in the BG group. Evodiamine (12.5 µg/mL) could significantly (*P*<0.01) down-regulate the ratio of LC3-II to β-actin as compared to the NC group. Additionally, evodiamine (12.5 µg/mL) also could significantly (*P*<0.01) inhibit the ratio of p62 (a well-known autophagic substrate) to β-actin as compared to the NC group (**Fig. 5** **D**).

**Figure 6:**
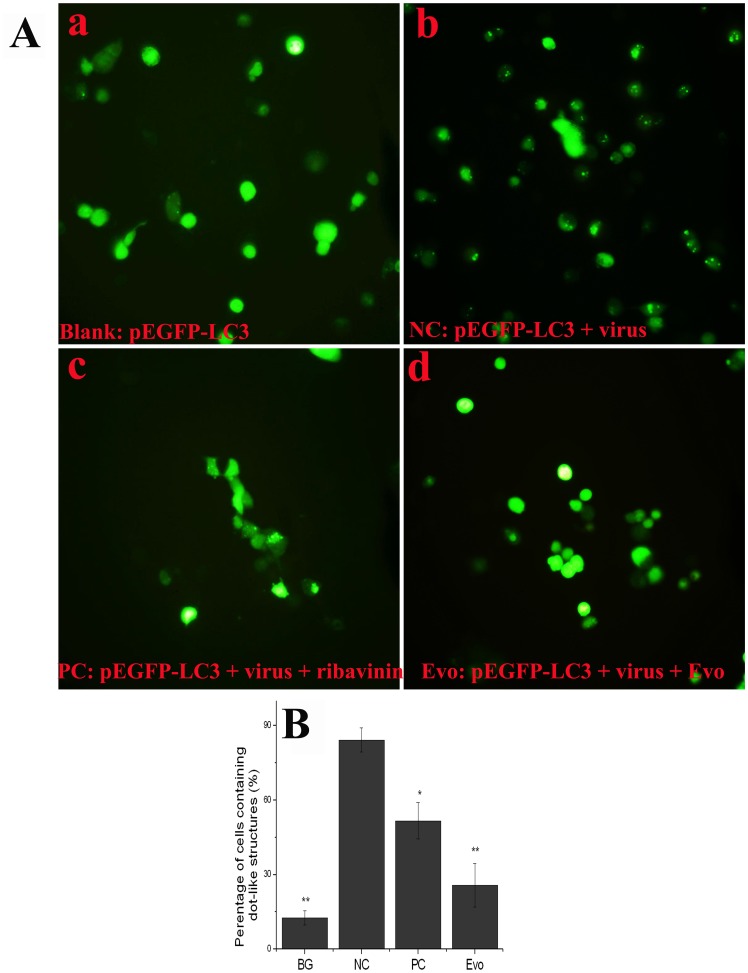
Evodiamine inhibits the IAV-induced autophagy determined by EGFP-LC3 assay. (**A**) Evodiamine inhibits the dot-like aggregation of EGFP-LC3 onto autophagosomes. (**B**) The percentage of cells containing EGFP-LC3 dots to cells expressing EGFP. After transfection with the pEGFP-LC3 plasmid for 6 h, the cells were infected with IAV (MOI = 2.0) and treated as **Fig. 5**. At 8 h post infection, the cells were visualized under an invert fluorescence microscope (10×40), the percentages of cells containing EGFP-LC3 dots to cells expressing EGFP were calculated in 10 fields chosen at random from three independent experiments in a 6-well microplate. Data shown were the mean±SD of three independent experiments. *P<0.05, **P<0.01, *vs*. the NC.

It is well known that LC3-II can accumulate on the autophagosome membrane. We constructed a fusion protein, EGFP-LC3, which could form dot-like structures on autophagosome membrane that were considered to be a specific marker of autophagosomes. As demonstrated in **Fig. 6** **A** and **B**, the percentage of cells containing EGFP-LC3 dots to cells expressing EGFP significantly (*P*<0.01) increased after virus infection (NC group) as compared with the blank group, whereas this percentage in the evodiamine-treatment (12.5 µg/mL) group significantly decreased as compared with the NC group. These findings indicated that evodiamine could inhibit the accumulation of autophagosome induced by IAV.

### Evodiamine Inhibits the Expressions of Atg5, Atg7 and Atg12

**Figure 7:**
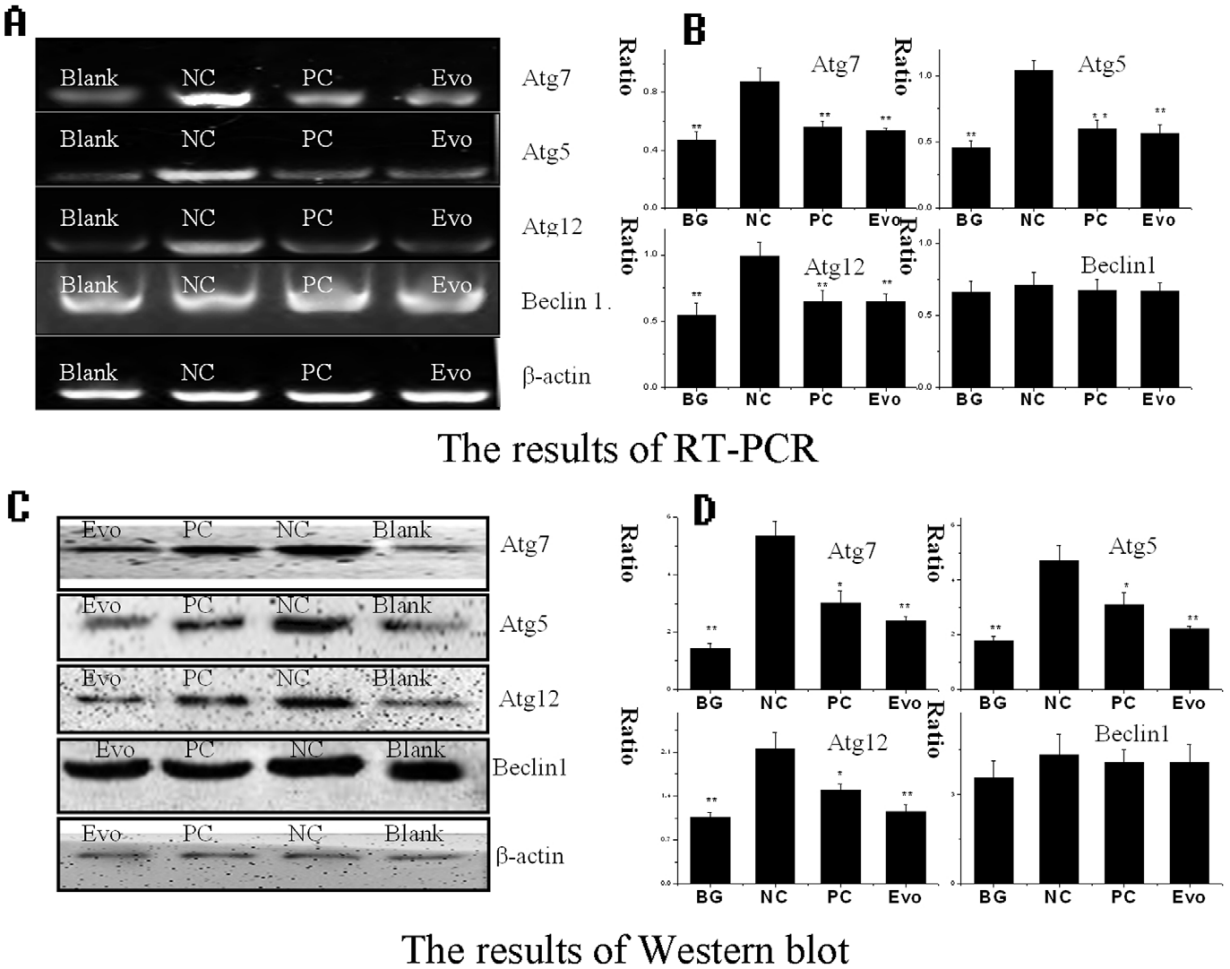
Evodiamine inhibits the expression of autophagy-related genes. (**A**, **B**) The results of the RT-PCR assay. (**C**, **D**) The results of the Western blot assay. In the BG group, the A549 cells were not infected with IAV and not treated with any drugs; In the NC group, the A549 cells were infected with IAV but not treated with drugs; In the PC and Evo groups, the A549 cells were infected with IAV and treated with ribavirin (25 µg/mL) and evodiamine (12.5 µg/mL), respectively. MOI = 0.001, incubation time = 24 h. The total gray value of each band was determined using a Gel-Pro analyzer 6.0. The data shown were the ratios of the expression of the target gene to β-action. Data shown were the mean±SD of three independent experiments. *P<0.05, **P<0.01, *vs*. the NC.

IAV infection can up-regulate the expression levels of Atg7, Atg16L1 and the Atg5-Atg12 heterodimer [19]. Here, we detected the influence of evodiamine on the expressions of Atg7, Atg5, Atg12 and Beclin1. As shown in **Fig. 7**, the expressions of Atg5, Atg7 and Atg12 in the NC group were significantly increased as compared to the blank group (BG). Ribavirin (25 µg/mL) and evodiamine (12.5 µg/mL) significantly inhibited the expressions of Atg5, Atg7 and Atg12 at both the mRNA transcription and protein translation levels as compared to the NC group. The influence of evodiamine on the expression of Beclin1 was not obvious.

### Evodiamine Inhibits IAV-induced Autophagy may be via AMPK/TSC2/mTOR Pathway

**Figure 8:**
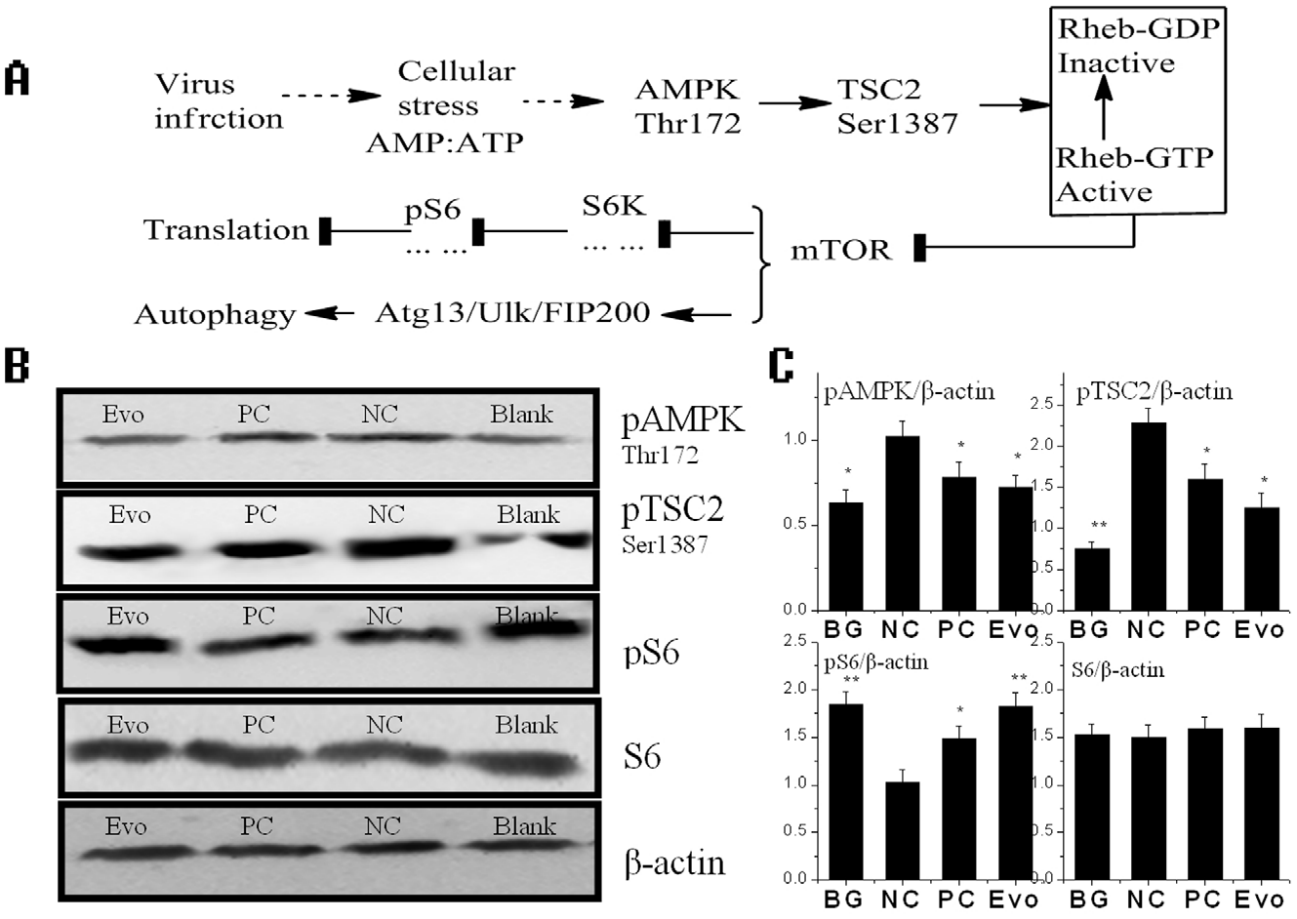
Evodiamine influences the AMPK/TSC2/mTOR pathway after IAV infection. (**A**) The speculated cellular response to viral infection mediated by AMPK/TSC2/mTOR pathway. (**B, C**) The influence of evodiamine on the AMPK/TSC2/mTOR signal pathway determined by Western blot assay. The treatments were same with that mentioned in **Fig. 7** The data shown were the ratios of the expression of the target gene to β-action. Data shown were the mean±SD of three independent experiments. *P<0.05, **P<0.01, *vs*. the NC.

Recently, Ma J. et al have shown that the highly pathogenic avian influenza viruses (H5N1) can cause “autophagic cell death” through suppressing mTOR signaling, which is dependent on TSC2 activity [7]. TSC2 can be regulated through differential phosphorylation mechanism, such as LKB1/AMPK, PI3K/AKT and p38 MAPK pathways. Here we examined the influence of evodiamine on the AMPK/TSC2/mTOR signal pathway in MDCK cells after IAV infection. As shown in **Fig. 8** **B** and **C**, pAMPK and pTSC2 was up-regulated after IAV infection (NC group) comparing with the blank group (BG), and evodiamine (Evo group) could inhibit the levels of pAMPK and pTSC2 comparing with the NC group. The phospho-S6 (pS6) is a marker of mTOR activation. After IAV infection (NC group), the pS6 was down-regulated comparing with the BG group, and evodiamine (Evo group) could increase pS6 comparing with the NC group. Ribavirin (25 µg/mL) also had little effect on the AMPK/TSC2/mTOR signal pathway.

### Evodiamine can Inhibit the Cytokine Release of TNF-α, IL-1β, IL-6 and IL-8 Induced by IAV

**Figure 9:**
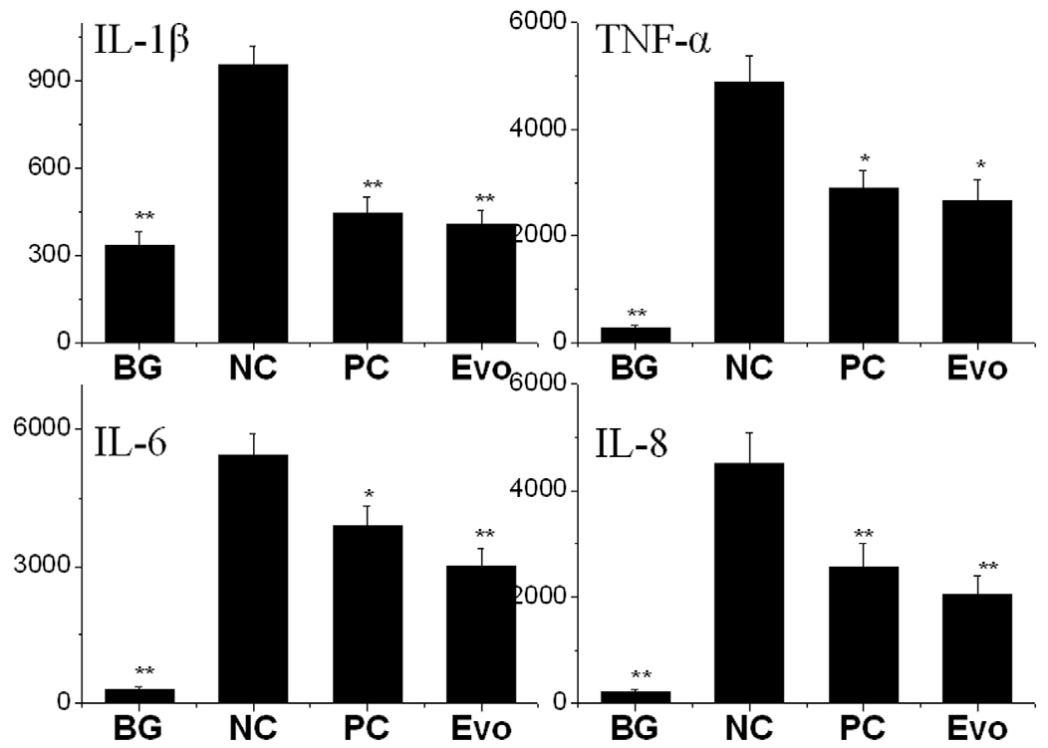
The influence of evodiamine on the cytokine release determined by ELISA (pg/mL). Cell culture supernatants were collected and frozen at −80°C. The treatments were same with that mentioned in **Fig. 7** Data shown were the mean±SD of three independent experiments. *P<0.05, **P<0.01, *vs*. the NC.

Autophagy can directly regulate the expression a number of inflammatory cytokines [20]. Here we also determined the influence of evodiamine on the release of TNF-α, IL-1β, IL-6 and IL-8 of A549 cell determined by ELISA. As shown in **Fig. 9**, after IAV infection, the levels of TNF-α, IL-1β, IL-6 and IL-8 were significantly (p<0.01) increased, whereas both of ribavirin (25 µg/mL) and evodiamine (12.5 µg/mL) significantly inhibited the release of TNF-α, IL-1β, IL-6 and IL-8.

## Discussion

Up to now, there are several anti-IAV drug screening methods have been reported. There are several virus-based drug screening models which target the neuraminidase [21], the M2 ion channel [22] and the RNA-dependent RNA polymerase [23], and there are also several cell-based drug screening models which target the cytopathic effect (CPE) [24], [25], [26]. Usually, virus-based drug screening models often lead to the inevitable selection of drug-resistant viral mutants, whereas cell-based drug screening models that target cell factors often decrease these resistant mutants [27]. It is well known that autophagy is a highly conserved process in all eukaryotic cells, so the drug screening method that we have established may lead to the discovery of novel antiviral drugs that resist the development of drug-resistant virus strains.

Although there are several cell-based drug screening models that target the CPE, we think these models have some limitations, because there is an effective antiviral mechanism used by the infected cells to commit suicide by triggering premature apoptosis [28], [29], and the antiviral mechanism of many drugs may work by promoting the apoptosis of virus-infected cells. Thus, after drug treatment, the CPE may be larger than that with no drug treatment. In our drug screening model, we detect the BiFC-FRET signal at 8 h p.i. (MOI = 2.0). The time of drug treatment is relatively short, before the CPE occurs, the drugs that inhibit IAV-induced autophagy can be picked out.

Using this drug screening method, we have obtained 20 examples of medicinal plants that can significantly (*P*<0.05 or 0.01) inhibit the formation of the Atg5-Atg12/Atg16 heterotrimer. After plaque inhibition assay, we find all of them have anti-IAV activity (Data not shown), which may prove that inhibiting the formation of the Atg5-Atg12/Atg16 heterotrimer can really inhibit the replication of IAV.

In these 20 medicinal plants, there are 7 examples that have not been reported to possess anti-IAV activity, the crude extract of *Evodia rutaecarpa* Benth shows the highest activity, so we choose *Evodia rutaecarpa* Benth as our medicinal plant of interest, and explore the mechanism of evodiamine, the major active component of *Evodia rutaecarpa* Benth, on anti-IAV activity. After a series of assays, we find evodiamine not only inhibits the formation of the Atg5-Atg12/Atg16 heterotrimer, but also inhibits the accumulation of LC3-II and p62, the dot-like aggregation of EGFP-LC3 and the expressions of Atg5, Atg7 and Atg12 after IAV infection.

Moreover we also have determined the influence of evodiamine on the AMPK/TSC2/mTOR signal pathway after IAV infection. As shown in **Fig. 8** **A**, AMPK is a cellular energy sensor. It is activated by the high ratio of AMP/ATP, which is indicated by the Thr172 phosphorylation in AMPK. AMPK activation causes the phosphorylation of Thr1227, Ser1387 and Ser1345 in TSC2 [11], [30], and enhances TSC2 activity. TSC2 is a GTPase-activating protein (GAP) toward Rheb, a Ras family GTPase. TSC2 inactivates Rheb by directly stimulating Rheb-GTP hydrolysis. Rheb-GTP activates mTOR by phosphorylating mTOR on Ser 2448 [31]. So AMPK activation can enhance TSC2 activity, promotes Rheb-GTP hydrolysis, then inhibits mTOR activity, and finally stimulates autophagy. Virus infection can lead to cellular stress, which may activate AMPK. Ma J. et al have shown that avian influenza A virus H5N1 can suppress mTOR signaling dependent on TSC1/TSC2 complex and induces autophagic cell death [7]. In our study, we have shown that the activities of AMPK and TSC2 are increased and the mTOR activity is suppressed after IAV infection, whereas evodiamine can reverse this process, and inhibits autophagy induced by IAV.

In addition, some scientists have suggested that the cytokine storm effects will contribute to the high death rate in a flu pandemic, autophagy may be responsible for the cytokine storm induced by the H5N1 and H9N2 viruses [32], Autophagy can positively regulate the transcription and secretion of TNF-α, IL-8 and IL-6. Disruption of normal autophagic pathways has been linked the inflammasome activation and the increased secretion of IL-1α and IL-1β [20]. Here we have shown evodiamine can inhibit the release of TNF-α, IL-1β, IL-6 and IL-8 after IAV infection, the underlying mechanism may be related to the inhibition of autophagy by evodiamine.

It has been reported that many viruses exploit the autophagy machinery to facilitate their replication, such as hepatitis B virus [33], hepatitis C virus [34] and coxsackievirus B3 [35]. Thus, drugs screened by our drug screening method may have the potential for a broad-spectrum of antiviral activity.

In conclusion, we have established a drug screening method based on the autophagy pathway utilizing BiFC-FRET technique, and show that evodiamine may be a promising new inhibitor of IAV infection.

## Materials and Methods

### Medicinal Plant and Compound

Medicinal plants were purchased from the Yulin medicine market (Guangxi, China). Specimens were deposited in our lab. Evodiamine (purity>98%) was purchased from the National Institutes for Food and Drug Control (Beijing, China).

### Viruses, Cells and Cytotoxicity Assay

The virus stocks of IAV subtypes A/ShanTou/169/06(H1N1), A/PuertoRico/8/34 (H1N1), A/ShanTou/1233/06 (H1N1), A/ShanTou/602/06 (H3N2), A/ShanTou/364/05 (H3N2), A/Quail/HongKong/G1/97 (H9N2), A/Chicken/Guangdong/A1/03 (H9N2) and A/Chicken/GD/1/05 (H5N1) were prepared in MDCK cells or 10-day-old embryonating eggs. The virus titer was determined by a plaque assay [36]. Except for the antiviral assay by the SRB method using CPE reduction, we only used A/ShanTou/169/06(H1N1) as the test virus strain in other experiments. The cytotoxicity of the test drugs on the MDCK and A549 cells was determined using a MTT assay, as previously reported [37]. The concentration of the test drugs required to lower cell viability by 50% (IC_50_) was calculated using Origin 8.0 software. The maximal concentration with no cytotoxicity was used as the optimal concentration.

### Construction of Plasmids

To construct the BiFC-FRET plasmids, N′- and C′- segments of a red fluorescent protein (GenBank:HQ423140.1), corresponding to amino acids 1 to 159 and 160 to 262, respectively, were inserted into pcDNA 3.0 and named pMC and pMN plasmids, respectively. Human atg5 (NM_004849.2) was inserted into pMC and named pMC-atg5. Human atg12 (NM_004707.3) was inserted into pMN and named pMN-atg12. Human atg16L1 (NM_030803.6) was inserted into pEGFP-C1 and named pEGFP-atg16. To construct the IAV vRNA promoter luciferase reporter plasmid, human pol-I promoter (GenBank:NT_167214.1) was cloned into a pGL3-basic vector and named pPol-1-p vector. The firefly luciferase (Luc) gene was cloned into the pPol-1-p vector and named pPolI-vluc, which contained a negative strand luciferase (-Luc) gene flanked by the 5′ and 3′ untranslated regions (UTRs) of the IAV A/PuertoRico/8/34 NP segment, and followed by a human pol-I terminator at 3′ untranslated regions. In addition, the human LC3B gene (NM_022818.4) was inserted into pEGFP-C1 and named pEGFP-LC3. All constructs were verified by DNA sequencing.

### Initial Screening Assay

In the initial screening, A549 cells were seeded into the 96-well microplate for 24 h, then cotransfected with pMC-atg5, pMN-atg12 and pEGFP-Atg16 plasmids using Lipofectamine 2000 (Invitrogen). After 6 h, the cells were infected with IAV (MOI = 2.0) and treated with drugs for 8 h. After 1 h at 4°C, the fluorescence intensity was determined using a Tecan infinite M1000 premium Quad4 monochromator microplate reader. To assess the interaction between Atg5-Atg12 heterodimer and Atg16L1 in the BiFC-FRET assay, the fluorescence intensities were determined at 610 nm and 509 nm with a 20-nm bandwidth after excitation at 488 nm with a 20-nm bandwidth, 100-µs lag time and 200-µs integration time. The FRET efficiency (FRET^e^) was expressed as the ratio of the acceptor (610 nm) and the donor (509 nm) emission intensities according to the following formula:
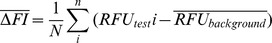


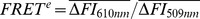


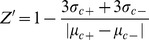



In these equations, “RFU*_test_*” was the fluorescence intensity of test groups, RFU*_background_* was the fluorescence intensity of background, RFU was the relative fluorescence unit. The Z′-factor was a statistical parameter to quantify the suitability of a particular assay for use in a high-throughput screen [18]. The notations σ_c+_ and σ_c−_ were the standard deviations of the negative control (NC) and the blank group (BG), respectively, and µ_c+_ and µ_c−_ were the average values of the NC and BG groups, respectively.

To observe the influence of our test drugs on the formation of the Atg5-Atg12/Atg16 heterotrimer, A549 cells were plated onto glass cover slips in 6-well plates that were coated with poly-L-lysine for 24 h and then were transfected using Lipofectamine 2000 (Invitrogen). After 6 h, the cells were infected with IAV (MOI = 2.0) and treated with drugs for 8 h. After 1 h at 4°C, the cells were fixed using 3% formaldehyde for 20 min and then visualized using an upright fluorescence microscope (Nikon Eclipse 90i). All images were acquired under the same setting.

### Plaque Inhibition Assay

A549 cells were seeded in a 6-well plate for 24 h, the viruses were pretreated with a medium containing evodiamine (1.54, 3.08, 6.25 and 12.5 µg/mL) for 2 h. After the A549 cells were washed with PBS 3 times, the pretreated viruses were added and adsorbed for 1 h (MOI = 0.001), washed with PBS 3 times, and then the cells were cultivated in the mediums containing evodiamine (1.54, 3.08, 6.25 and 12.5 µg/mL) for 48 h. After frost-thawing one time, the supernatants were collected, and the titers were determined with a standard plaque assay [36]. The concentration of compound required to inhibit the virus titer by 50% (50% effective concentrations [EC_50_]) was calculated using Origin 8.0 software. The antiviral index was represented as the ratio of the IC_50_ to EC_50_.

### IAV vRNA Promoter Luciferase Assay

A549 cells were seeded in a 24-well plate for 24 h. After transfection with the pPolI-vluc plasmid for 6 h, IAV and the test drug were added (MOI = 0.01), and the cell lysate was collected at 18 h post infects (p.i.). Luciferase activity was determined following the instrument of Luciferase Reporter Assay Kit (BD Biosciences Clontech).

### TCID50 and Antiviral Assay by the SRB Method using CPE Reduction

IAV stock solution was diluted with MEM medium in serial dilutions, after incubation with MDCK cells for 48 h, the TCID_50_ was calculated following the method of Reed and Muench. Antiviral activities of test drugs were evaluated by the sulforhodamine B (SRB) method using CPE reduction [38]. Briefly, MDCK cells were seeded in 96-well plate. 0.09 ml of virus suspension (50 TCID_50_) and 0.01 ml medium containing various concentrations of test compounds were added. At 48 h, after washing, 100 µl−20°C 70% acetone was added. After removing acetone, the plates were dried, and added 100 µl 0.4% (w/v) SRB, after washing, the plates were dried and added 100 µl 10 mM Tris-base solution. OD was read at 562 nm. Three wells were used each for the negative (virus-infected non-drug-treated) and the mock controls (non-infected non-drug-treated). 0.5%DMSO was used in each group. Percent protection of test compounds (cell viability) was calculated as following, the concentration of 50% protection was defined as the EC_50_.
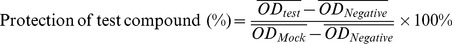



### Analysis of EGFP-LC3 Puncta

After transfection with the pEGFP-LC3 plasmid for 6 h, A549 cells were infected with IAV (MOI = 2.0) and treated with our drugs for 8 h. The cells were visualized under an invert fluorescence microscope (10×40), and the percentages of cells containing EGFP-LC3 dots to cells expressing EGFP were calculated in 10 fields chosen at random from three independent experiments.

### Reverse-transcription (RT-PCR)

Extraction of RNA and the RT-PCR reactions were performed according to the protocols of the TRIzol® Reagent Kit and the RT-PCR Kit (invitrogen). PCR products were electrophoresed in a 1% agarose gel and visualized on an UV-transilluminator.

### Western Blotting

Anti-LC3B, anti-Beclin1, anti-Atg5, anti-Atg7, anti-Atg12, anti-M2, anti-pAMPK(Thr172), anti-pTSC2(Ser1387) and anti-β-actin antibodies were purchased from Cell Signaling Technology® Inc Company. Anti-pS6 (Ser235/236) and S6 antibody were purchased from Santa Cruz Biotechnology Inc., USA. The Western blotting assay was performed as previously reported [39].

### Co-Immunoprecipitation Assay

To detect the influence of evodiamine on the interaction between the Atg5-Atg12 heterodimer and Atg16 in physiological conditions, A549 cells were seeded into a 6-well plate for 24 h, then cotransfected with the pMC-atg5, pMN-atg12 and pEGFP-atg16 plasmids, infected with IAV (MOI = 0.001) and treated with evodiamine (12.5 µg/mL), and the cell lysate was collected at 24 h p.i. The interactions were determined following the instrument of the Co-Immunoprecipitation Kit (Thermo scientific, #23600). Normal rabbit IgG was used as a control. The levels of Atg5, Atg16 and β-actin of the whole cell lysate were determined by Western blotting.

### Cytokines Secretion

Cell culture supernatants were collected and frozen at −80°C. Cytokines were quantified by specific ELISA Kits (R&D Systems GmbH, Wiesbaden, Germany) following the manufacturer’s instructions.

### Statistical Analysis

Data shown were the mean±SD. A statistical significance was determined using the SPSS 13.0 software.

## Supporting Information

Figure S1 **IAV interferes in the autophagy signal pathway.** Autophagy can be divided into several stages. During the elongation stage, Atg5 must conjugate with Atg12, mediated by Atg7 and Atg10. Subsequently, the Atg5-Atg12 heterodimer must conjugate with Atg16. The Atg5-Atg12/Atg16 heterotrimer can promote the transformation of LC3-I (Atg8) to LC3-II (Atg8-PE). This process can be reversed by Atg4, which promotes the conversion of LC3-II to LC3-I. LC3-II is necessary for the formation of autophagosome. During the maturation stage, autophagosome must fuse with lysosome, mediated by Beclin1. IAV involves in autophagy by 1) producing ROS, which damages Atg4, impedes the conversion of LC3-II (Atg8-PE) to LC3-I (Atg8), and leads to the increase of LC3-II; 2) binding to Beclin1 by the IAV M2 protein; and 3) up-regulating the expression of several autophagy related genes, which can increase the autophagic flux.
(TIF)

Figure S2 **The design of our drug screening method based on the BiFC-FRET technique.** The fluorescent intensity was determined at 610 nm and 509 nm after excitation at 488 nm using a microplate reader in a 96-well plate, the FRET efficiency (FRET^e^) was expressed as the ratio of acceptor (610 nm)- and the donor (509 nm)-emission intensities after the deduction of the background intensity.
(TIF)

Table S1 **Inhibition of plant extracts on the formation of the Atg5-Atg12/Atg16 heterotrimer.** Data shown are means±SD from three independent experiments performed in triplicate. *P<0.05, **P<0.01 *vs.* the NC.
(DOC)
