# Supplementary figures and images for: A Drug Screening Method Based on the Autophagy Pathway and Studies of the Mechanism of Evodiamine against Influenza A Virus
Source: PLoS One. 2012 Aug 10;7(8):e42706. doi: 10.1371/journal.pone.0042706 (PMC3416798; doi:10.1371/journal.pone.0042706)

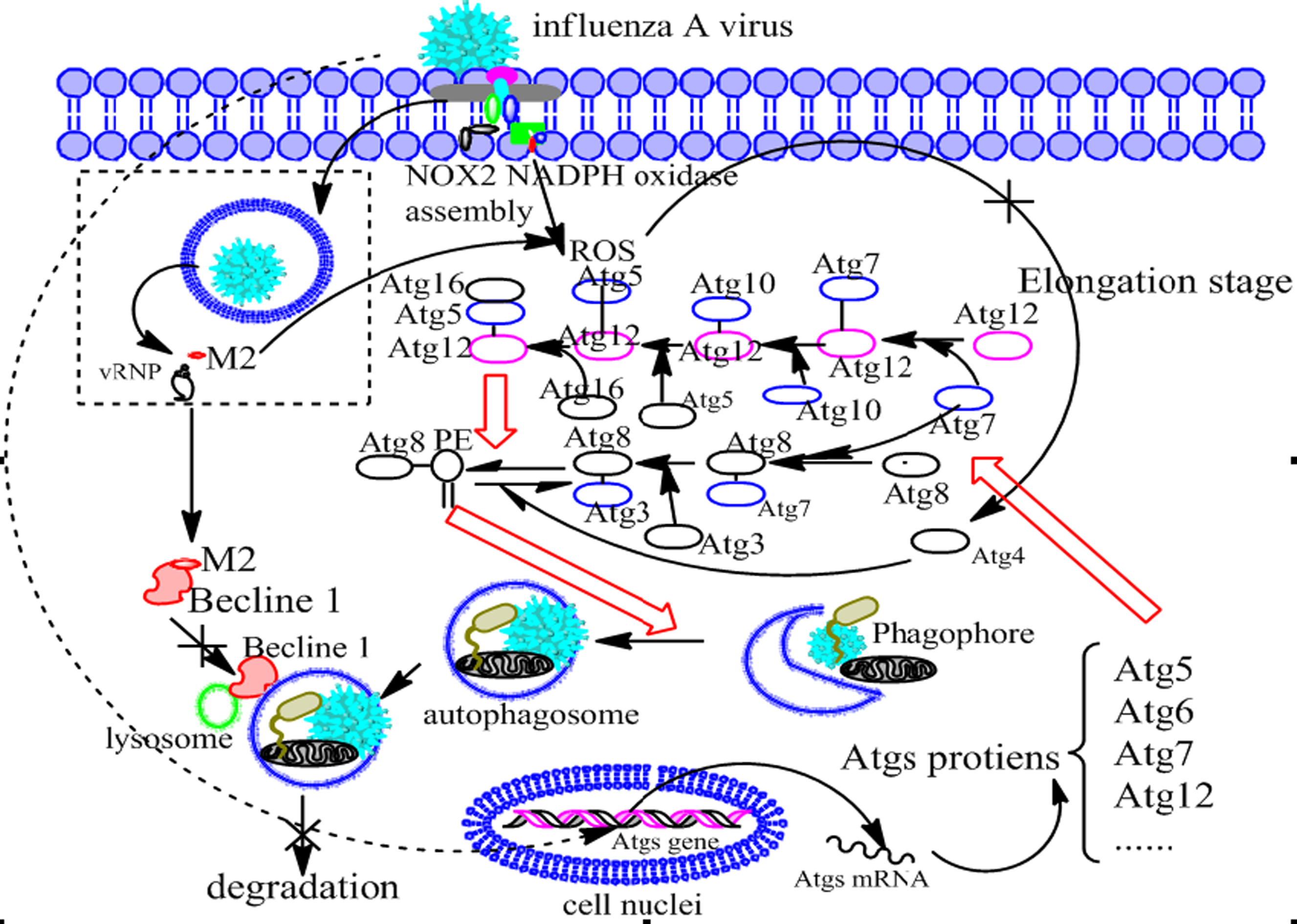

Supplement: Figure S1 — IAV interferes in the autophagy signal pathway. Autophagy can be divided into several stages. During the elongation stage, Atg5 must conjugate with Atg12, mediated by Atg7 and Atg10. Subsequently, the Atg5-Atg12 heterodimer must conjugate with Atg16. The Atg5-Atg12/Atg16 heterotrimer can promote the transformation of LC3-I (Atg8) to LC3-II (Atg8-PE). This process can be reversed by Atg4, which promotes the conversion of LC3-II to LC3-I. LC3-II is necessary for the formation of autophagosome. During the maturation stage, autophagosome must fuse with lysosome, mediated by Beclin1. IAV involves in autophagy by 1) producing ROS, which damages Atg4, impedes the conversion of LC3-II (Atg8-PE) to LC3-I (Atg8), and leads to the increase of LC3-II; 2) binding to Beclin1 by the IAV M2 protein; and 3) up-regulating the expression of several autophagy related genes, which can increase the autophagic flux. (TIF) [file pone.0042706.s001.tif]

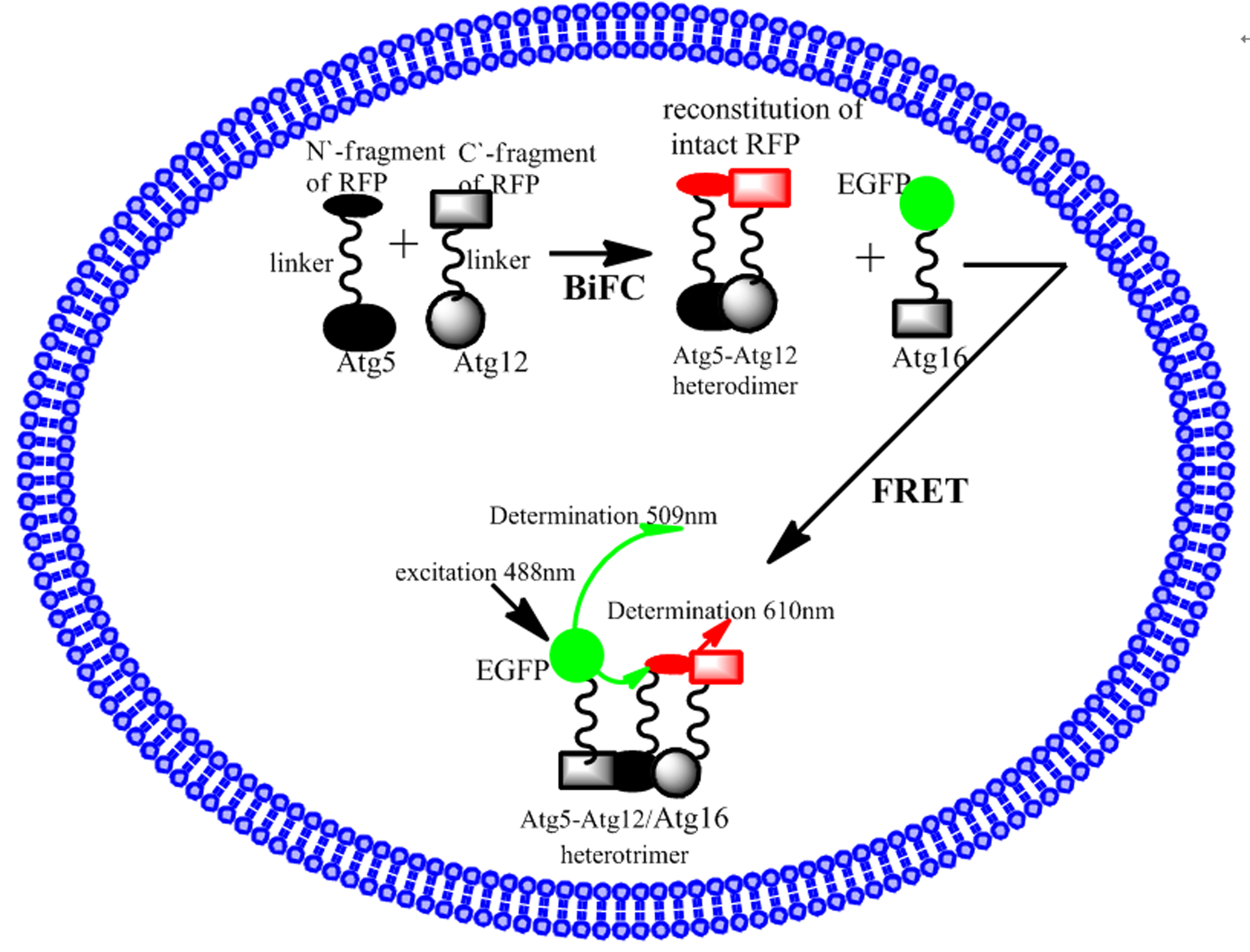

Supplement: Figure S2 — The design of our drug screening method based on the BiFC-FRET technique. The fluorescent intensity was determined at 610 nm and 509 nm after excitation at 488 nm using a microplate reader in a 96-well plate, the FRET efficiency (FRETe) was expressed as the ratio of acceptor (610 nm)- and the donor (509 nm)-emission intensities after the deduction of the background intensity. (TIF) [file pone.0042706.s002.tif]
