# Supplementary material for: A Drug Screening Method Based on the Autophagy Pathway and Studies of the Mechanism of Evodiamine against Influenza A Virus
Source: PLoS One. 2012 Aug 10;7(8):e42706. doi: 10.1371/journal.pone.0042706 (PMC3416798; doi:10.1371/journal.pone.0042706)
Supplement: Table S1 — Inhibition of plant extracts on the formation of the Atg5-Atg12/Atg16 heterotrimer. Data shown are means±SD from three independent experiments performed in triplicate. *P<0.05, **P<0.01 vs. the NC. (DOC) [file pone.0042706.s003.doc]

**Table S1 Inhibition of plant extracts on the formation of the Atg5-Atg12/Atg16 heterotrimer**

| Medicinal plants | Ratio 610nm/509nm | Medicinal plants | Ratio 610nm/509nm |
| --- | --- | --- | --- |
| Blank group (BG) | 0.2775 ± 0.0311** | Negative control group (NC) | 1.2753 ± 0.0978 |
| *Hibiscus mutabilis* L. | 1.1185 ± 0.2664 | *Arctostaphylos uva-ursi* subsp. *cratericola* | 0.8892 ± 0.1175 |
| *Stemona sessilifolia* (Miq.) Miq. | 0.9932 ± 0.1012 | *Cyperus rotundus* L. | 0.7996 ± 0.0775 |
| *Astragalus membranaceus* (Fisch.) Bge. | 0.8970 ± 0.0976 | *Salvia miltiorrhiza* Bge. | 0.8475 ± 0.0973 |
| *Genista tinctoria* Linn. | 1.1558 ± 0.1157 | *Radix Semiaquilegiae* | 0.9740 ± 0.0995 |
| *Sophora subprostrala* Chun et T.Chen. | 0.9936 ± 0.0883 | *Bambusa tuldoides* Munro. | 0.7887 ± 0.0965 |
| *Arctium lappa* L. | 0.8129 ± 0.0559 | *Glechoma hederacea* L. | 1.0776 ± 0.0886 |
| *Mentha haplocalyx* Briq. | 1.0837 ± 0.0721 | *Rohdea japonica* Roth. | 0.7758 ± 0.0841 |
| *Saposhnikovia divaricata* (Turcz.) Schischk. | 0.7477 ± 0.0445 | *Euphorbia hirta .* L. | 0.8768 ± 0.0674 |
| *Artemisia argyi* Levl. et Vant. | 0.7666 ± 0.0875 | *Xanthium sibiricun* Patr. | 0.9456 ± 0.0765 |
| *Kaempferia galanga* L. | 0.9065 ± 0.0829 | *Bos taurus* domesticus Gmelin. | 0.9938 ± 0.0987 |
| *Pogostemon cablin* (Blanco) Benth. | 0.8869 ± 0.0756 | *Bolbostemma  paniculatum*(Maxim.) Franquet. | 0.8998 ± 0.0858 |
| *Citrus reticulate* Blanco. | 0.9972 ± 0.0885 | *Trichosanthes Kirilowii* Maxim. | 0.6635 ± 0.0845 |
| *Paeonia lactiflora* Pall. | 0.8495 ± 0.0976 | *Vitex trifolia* L. | 0.8876 ± 0.0988 |
| *Indigo naturalis* | 0.7669 ± 0.0945 | *Equisetum hiemale* L. | 0.7989 ± 0.0979 |
| *Andrographis paniculata* (Burm. F. ) Nees. | 0. 7659 ± 0.0733 | *Erodium stephanianum* Willd. | 0.6756 ± 0.0658 |
| *Pogostemon cablin* (Blanco) Benth. | 0.7597 ± 0.0968 | *Siegesbeckia orientalis* L. | 0.6867 ± 0.0956 |
| *Anglica dahurica* (Fich. Ex Hoffm.) Benth.ex Hook | 0.8876 ± 0.0868 | *Punica granatum* L. | 0.5847±0.0736 |
| *Smilax glabra* Roxb. | 0.7983 ± 0.0943 | *Eucommia ulmoides* Oliv. | 0.7464 ± 0.0895 |
| *Scutellaria barbata* D. Don. | 0.7865 ± 0.0778 | *Pulsatilla chinensis* (Bge.)Reg. | 0.7633 ± 0.0988 |
| *Glycyrrhiza uralensis* Fisch. | 0.7688 ± 0.0889 | *Fraxinus rhynchophylla* Hance. | 0.6698 ± 0.0575 |
| *Polygonum cuspidatum* Sieb. et Zucc. | 0.8883 ± 0.0843 | *Glycine max*(L.)　Merr. | 0.8895 ± 0.0799 |
| Curcuma phaeocaulis Val. | 0.7793 ± 0.0884 | Polygonatum sibiricum Red. | 0.7938 ± 0.0917 |
| Mahonia fortunei (Lindl.)Fedde | 0.9910 ± 0.0776 | Pinus tabulaefomis Carr. | 0.9176 ± 0.0856 |
| Morus alba L. | 0.8747 ± 0.0865 | Chaenomeles speciosa (Sweet) Nakai | 0.6544 ± 0.0687 |
| *Aristolochia debilis* Seib.et Zucc. | 0.9059 ± 0.1724 | *Achyranthes bidentata* Bl*.* | 0.7947 ± 0.0857 |
| *Reseda odorata* L. | 0.6466 ± 0.0998 | *Platycladus orientalis* (L.) Franco. | 0.7756 ± 0.0984 |
| *Kaempferia galanga* L. | 0.8928 ± 0.0793 | *Panax notoginseng* (Burt.) F. H. Chen. | 0.7846 ± 0.0874 |
| *Acacia catechu* (L.F.) Willd. | 0.8959 ± 0.0998 | *Rbia cordifolia* L. | 0.9647 ± 0.0798 |
| *Lantana camara* L. | 0.7068 ± 0.8176 | *Sanhuisorba officinalis* L. | 0.7467 ± 0.0766 |
| *Sophora flavescens* Ait. | 0.7785 ± 0.0556 | *Hypericum japonicum* Thunb. | 0.7608 ± 0.0727 |
| *Verbena officinalis* L. | 0.7649 ± 0.8488 | *Patrinia scabiosaefolia* Fisch. | 0.7988 ± 0.0446 |
| *Serissa serissoides* (DC.) Druce | 0.8174 ± 0.0665* | *Dryopteris setosa* (Thunb.) Akasawa | 0.7654 ± 0.0710 |
| *Peucedanum praeruptorum* Dunn. | 0.5374 ± 0.0464* | *Aloe barbadensis* Miller. | 0.6148 ± 0.0573* |
| *Borneolum syntheticum* | 0.6598 ± 0.0439* | *Ginkgo biloba* L. | 0.4685 ± 0.0687* |
| *Schizonepeta tenuifolia* Briq. | 0.6689 ± 0.0991* | *Hedyotis diffusa* Willd. | 0.5975 ± 0.0418* |
| *Rheum palmatum* L. | 0.5598 ± 0.0576* | *Litsea cubeba (Lour.)* Pers. | 0.4966 ± 0.0758* |
| *Kummerowia striata* (Thunb.) Schindl | 0.5947 ± 0.0455* | *Apium graveolens* L. var. dulce DC. | 0.4858 ± 0.0574* |
| *Chrysanthemum indicum* L. | 0.5473 ± 0.0332* | *Scutellaria baicalensis* Georgi. | 0.6643 ± 0.0573* |
| *Coptis chinensis* Franch. | 0.5980 ± 0.0289* | *Silybum marianum* L. | 0.5648 ± 0.0771* |
| *Areca catechu* L. | 0.5761 ± 0.0662** | *Eugenia caryophyllata* Thunb. | 0.3747 ± 0.0486** |
| *Buddleja lindleyana* Fort. | 0.4986 ± 0.0338** | *Curcuma longa* L*.* | 0.4381 ± 0.0338** |
| ***Evodia rutaecarpa* Benth.** | 0.3087 ± 0.0662** |  |  |

Data shown are means ± SD from three independent experiments performed in triplicate.* *P* < 0.05, ** *P* < 0.01 *vs.* the NC.
